# Organ Preservation for Advanced Laryngeal Cancer: Experience with Concurrent Chemoradiation Therapy

**DOI:** 10.7759/cureus.7553

**Published:** 2020-04-06

**Authors:** Asif Ali Arain, Muhammad Shaheryar Ahmed Rajput, Shabbir Akhtar, Arsalan A Rajput, Mohammad Adeel, Ahmad Hatem, Ahmed Nadeem Abbasi

**Affiliations:** 1 Otolaryngology, The Indus Hospital, Karachi, PAK; 2 Otolaryngology, Liaquat University of Medical and Health Sciences, Jamshoro, PAK; 3 Otolaryngology and Head and Neck Surgery, Aga Khan University Hospital, Karachi, PAK; 4 Otolaryngology and Head and Neck Surgery, King Faisal Specialist Hospital and Research Centre, Riyadh, SAU; 5 Surgery, Aga Khan University, Karachi, PAK; 6 Ophthalmology, Aga Khan University Hospital, Karachi, PAK; 7 Otolaryngology, Bradford Royal Infirmary, Bradford, GBR; 8 Otolaryngology, Shaukat Khanum Memorial Cancer Hospital and Research Centre, Lahore, PAK; 9 Head and Neck Oncology, Sheffield Teaching Hospitals National Health Service Foundation Trust, Sheffield, GBR; 10 Otolaryngology, Head and Neck Surgery, King Faisal Specialist Hospital and Research Centre, Riyadh, SAU; 11 Oncology, Aga Khan University Hospital, Karachi, PAK

**Keywords:** laryngeal carcinoma, organ preservation, concurrent chemoradiation therapy, ccrt, ic, rt, lfs

## Abstract

Introduction

The larynx is a part of the upper respiratory tract that performs many essential functions including breathing, speaking, and swallowing. For this reason, the quality of life is significantly affected by laryngeal cancer and its treatment. Therefore, the focus of management for the last few decades has been on preserving the function of a larynx without compromising survival. This study was done with the purpose of reviewing our experience of organ preservation approach with concurrent chemoradiation therapy (CCRT) for locally advanced cancers of larynx.

Methods

A retrospective chart review was carried out for the data of pathology reports and clinical notes of the patients who were diagnosed with laryngeal squamous cell carcinoma and primarily treated with CCRT at our tertiary care institute from November 2010 to June 2015.

Results

Of 25 patients included in the study, there were 19 males and six females. The mean age was 56 years. On comparison of post-treatment CT scan following eight weeks of completion of therapy, 21 patients showed complete resolution of the disease and four patients had persistent disease who were later treated with salvage laryngectomy. The speech was understandable in 18 patients and poor or not understandable in seven patients. Three patients had chronic aspiration and breathing difficulties necessitating permanent tracheostomy. Three patients required permanent gastrostomy due to chronic dysphagia, one of them belonged to those who were also tracheostomized.

Conclusions

Our experience with CCRT as an organ preservation approach for advanced laryngeal cancers was promising. When considering the functional organ preservation, the proportion of success is remarkably less; however, the overall impression is worthy enough to uphold the sentiment in favor of non-surgical organ preservation. The debate is ongoing in the quest of finding a balanced approach with acceptable toxicity and decent functional outcome with adequate speech, breathing, and swallowing.

## Introduction

Cancer is one of the leading causes of death and forms the single most important barrier to improve life expectancy worldwide in the era of modern medicine [1]. The reasons for rapidly growing cancer incidence and mortality are complex and probably reflect the increasing world population and aging. Decline in mortality rates of stroke and ischemic heart disease also partly responsible for this phenomenon of cancer emerging as a leading cause of death in many countries [2,3]. This transition seems to be more dramatic in developing countries [1].

The larynx is a part of the upper respiratory tract that performs many essential functions including breathing, speaking, and swallowing. For this reason, the quality of life is significantly affected by laryngeal cancer and its treatment. Therefore, the focus of management for the last few decades has been on preserving the function of the larynx without compromising survival [4]. 

The three landmark studies published in 1991, 1996, and 2003 investigated and evaluated locally advanced laryngeal and hypopharyngeal cancers [5-7]. In 2006, a multidisciplinary expert panel reviewed the literature available through 2005 and the results of these landmark studies led to the development of clinical practice guidelines for the use of larynx-preservation strategies by the American Society of Clinical Oncology (ASCO). ASCO concluded the review with the following recommendations: “All patients with T1 or T2 laryngeal cancer, with rare exception, should be treated initially with intent to preserve the larynx. For most patients with T3 or T4 disease without tumor invasion through cartilage into soft tissues, a larynx-preservation approach is an appropriate, standard treatment option, and concurrent chemoradiation therapy is the most widely applicable approach. To ensure an optimum outcome, special expertise and a multidisciplinary team are necessary, and the team should fully discuss with the patient the advantages and disadvantages of larynx-preservation options compared with treatments that include total laryngectomy” [8].

These guidelines published by ASCO in 2006 form the basis of the current standard of practice in the treatment of laryngeal cancer to avoid total laryngectomy as the preferred treatment option. This has been reconfirmed in the number of recent reviews [9-12]. Total laryngectomy is generally recognized as one of the radical and mutilating surgical procedures most dreaded by patients. Common consequences following total laryngectomy include social isolation, job loss, and depression [8]. Although the non-surgical management has largely replaced surgery for a selected group of advanced laryngeal carcinoma, there still remains an important role of surgery, in particular, total laryngectomy in cases of advanced extra laryngeal and recurrent disease [13]. This study was done with the purpose of reviewing our experience of the organ preservation approach with concurrent chemoradiation therapy (CCRT) for locally advanced cancers of larynx.

## Materials and methods

Institutional ethical approval was obtained before starting the study. A retrospective chart review was carried out for the data of pathology reports and clinical notes of the patients who were diagnosed with laryngeal squamous cell carcinoma and primarily treated with CCRT at our tertiary care institute from November 2010 to June 2015.

Patients with a history of previous treatment including surgery, radiotherapy, and chemotherapy were all excluded. A total of 25 patients were included in the study with the diagnosis of locally advanced laryngeal carcinoma. These patients were offered chemoradiation therapy as a protocol for organ preservation being in practice during the given period at the institute after full discussion with the patient and the family about the pros and cons of larynx preservation versus total laryngectomy.

The staging was done according to the seventh edition of the AJCC (American Joint Commission on Cancer) [14]. Larynx and upper neck were treated with 2.0 Gy per fraction, once a day, for five days a week to a total dose of 70 Gy in 35 fractions over a period of seven weeks. Concurrent chemotherapy with cisplatin or carboplatin was given as a three-weekly schedule (days 1, 22, 43).

Treatment response was assessed by comparison of pre- and post-treatment CT scans with contrast. Post-treatment CT scan with contrast was done after eight weeks of completion of therapy. The functional outcome of larynx preservation was assessed for the speech, need of gastrostomy tube, and tracheostomy tube. Statistical package for social sciences version 18 (SPSS Inc., Chicago, IL) was used for statistical analysis.

## Results

Of 25 patients included in the study, there were 19 males and six females. The mean age was 56 years. All the patients had stage III disease. The tumor was limited to the larynx in 23 patients and in the other two patients, the pyriform fossa was involved. These variables are shown in Table 1.

**Table 1:** Gender, age, and staging

Variables		Results
Gender	Male	19
	Female	6
Mean age		56 years
Age range		46-72 years
Disease extent	Stage III, limited to larynx	23
	Stage III, pyriform fossa involved	2

Histologically, 12 of 25 patients had grade I, nine had grade II and four had grade III tumors as shown in Table 2.

**Table 2:** Histological grading of tumors SCC: squamous cell carcinoma

Tumor grade	Histology	Number of patients
Grade I	Well differentiated SCC	12
Grade II	Moderately differentiated SCC	9
Grade III	Poorly differentiated SCC	4

On comparison of post-treatment CT scan following eight weeks of completion of therapy, 21 patients showed complete resolution of the disease and four patients had persistent disease who were later treated with salvage laryngectomy. Treatment response to CCRT is shown in Table 3.

**Table 3:** Treatment response to concurrent chemoradiation therapy

Treatment response	Number of patients
Complete resolution	21
Persistent disease	4

The speech was understandable in 18 patients and poor or not understandable in seven patients. Three patients had chronic aspiration and breathing difficulties necessitating permanent tracheostomy. Three patients required permanent gastrostomy due to chronic dysphagia, one of them belonged to those who were also tracheostomized. The total number of organs preserved was 21 (84%); however, nine organs were preserved with good function (36%). Organ and function preservation is shown in Table 4.

**Table 4:** Organ and function preservation

Outcomes		Number of patients
Speech	Understandable	18
	Poor	7
Breathing	Normal breathing	22
	Tracheostomy dependent	3
Swallowing	Able to swallow	22
	Gastrostomy dependent	3
Organ preservation	Total organs preserved	21
	Organ preservation with good function	9

## Discussion

The study on patient preference for longevity and voice preservation in 1981 revealed that 20% of the study population were willing to accept 20% to 30% less survival by opting for radiotherapy instead of total laryngectomy. This attitude was due to the fact that radiation therapy (RT) preserves normal or nearly normal speech [15]. However, the subjects in the study were all healthy individuals and their attitude may not be a true reflection of the feelings and emotions of a genuine patient.

The landmark publication by Veterans Affairs (VA) Laryngeal Cancer Study Group reported that the efficacy of chemotherapy followed by radiotherapy was similar to that of surgery followed by radiotherapy and offered the added benefit of laryngeal preservation in two-thirds of the patients treated by this approach [5]. However, in this study, organ preservation was interpreted more like an “organ in place” regardless of its functional capability.

Subsequently, Forastiere et al. (RTOG 91-11 trial) reported a randomized trial in which induction chemotherapy followed by radiotherapy was compared with two other regimens: concurrent chemoradiation therapy in one group and radiation therapy alone in another group of patients. The trial confirmed that the outcome in patients who were able to tolerate chemotherapy was best with CCRT [7]. However, there was no difference in overall survival amongst patients treated with other regimens. Similar to the study by VA, this trial was also lacking the reporting of laryngeal functions (swallowing, speaking, and breathing) which is necessary for a practical interpretation of the outcome.

The main disadvantage of organ preservation by CRT is the persistence of disease after completion of treatment or early relapse after curative treatment requiring salvage surgery [16]. Most of the surgeons worldwide accept salvage total laryngectomy as a suitable option after failure or recurrence for patients treated with organ preservation protocol. However, these patients are more prone to develop a number of complications as compared to those who were treated primarily with surgery [17-22].

The authors of the globally most noticeable RTOG 91-11 trial reported their long-term results in 2013 and essentially maintained their initial recommendations and concluded that 10-year results of induction chemotherapy followed by RT and CCRT were similar in efficacy for the laryngectomy-free survival (LFS) endpoint. However, locoregional control and larynx preservation were significantly improved with CCRT compared with the induction arm or RT alone. They recommended that new strategies to improve organ preservation and function with less morbidity are needed [23]. The CCRT offers a significantly higher chance of larynx preservation than RT alone or induction chemotherapy followed by RT, albeit at the cost of more acute toxicities and no improvement in overall survival [16]. Moreover, the CCRT arm showed lower compliance and a much higher rate of “late death unrelated to cancer” (36% in the CCRT arm vs. 18% in both comparator arms, RT or IC+RT) [16]. 

Lately, the German multicenter randomized phase II trial for larynx preservation for locally advanced laryngeal/hypopharyngeal cancer was published in the official journal of the European Society for Medical Oncology. The purpose of the study was to test the hypothesis that the addition of cetuximab to induction chemotherapy (IC) and radiotherapy improves LFS. Response to first IC cycle (IC-1) with >30% endoscopically estimated tumor surface shrinkage was used to define early responders; early salvage TL was recommended to non-responders [24]. This empirical endoscopically assessed cut-off of >30% was introduced the first time in literature. The primary objective of 24-month LFS of >35% was achieved in both arms of the study, 46.6% in arm A and 45.4% in arm B.

The endoscopic assessment of tumor is one of the routine ENT (ear, nose and throat) procedures and certainly provides a high level of assessment due to direct visualization than the comparison on imaging studies which likely be misleading in the first week of therapy. Consequently, non-responders can undergo early salvage total laryngectomy before completing the full course and therefore avoid higher toxicity of CCRT and complication rate of traditional late salvage laryngectomy.

## Conclusions

Our experience with CCRT as an organ preservation approach for advanced laryngeal cancers was promising. When considering the functional organ preservation, the proportion of success is remarkably less; however, the overall impression is worthy enough to uphold the sentiment in favor of non-surgical organ preservation. The debate is ongoing in the quest of finding a balanced approach with acceptable toxicity and decent functional outcome with adequate speech, breathing, and swallowing.
